# HIF-1α is a key mediator of the lung inflammatory potential of lithium-ion battery particles

**DOI:** 10.1186/s12989-019-0319-z

**Published:** 2019-09-18

**Authors:** Violaine Sironval, Mihaly Palmai-Pallag, Rita Vanbever, François Huaux, Jorge Mejia, Stéphane Lucas, Dominique Lison, Sybille van den Brule

**Affiliations:** 1 Louvain centre for Toxicology and Applied Pharmacology, Institut de Recherche Expérimentale et Clinique, Université catholique de Louvain, Avenue Hippocrate 57 - bte B1.57.06, 1200 Brussels, Belgium; 2 Louvain Drug Research Institute, Université catholique de Louvain, Avenue Mounier 73 - bte B1.73.12, 1200 Brussels, Belgium; 3 Research Centre for the Physics of Matter and Radiation (PMR-LARN), NARILIS, Université de Namur, rue de Bruxelles 61, 5000 Namur, Belgium

**Keywords:** Predictive toxicology, IL-1β, Epithelial cells, Biomarker, Cobalt, Nickel

## Abstract

**Background:**

Li-ion batteries (LIB) are increasingly used worldwide. They are made of low solubility micrometric particles, implying a potential for inhalation toxicity in occupational settings and possibly for consumers. LiCoO_2_ (LCO), one of the most used cathode material, induces inflammatory and fibrotic lung responses in mice. LCO also stabilizes hypoxia-inducible factor (HIF) -1α, a factor implicated in inflammation, fibrosis and carcinogenicity. Here, we investigated the role of cobalt, nickel and HIF-1α as determinants of toxicity, and evaluated their predictive value for the lung toxicity of LIB particles in in vitro assays.

**Results:**

By testing a set of 5 selected LIB particles (LCO, LiNiMnCoO_2_, LiNiCoAlO_2_) with different cobalt and nickel contents, we found a positive correlation between their in vivo lung inflammatory activity, and (i) Co and Ni particle content and their bioaccessibility and (ii) the stabilization of HIF-1α in the lung. Inhibition of HIF-1α with chetomin or PX-478 blunted the lung inflammatory response to LCO in mice. In IL-1β deficient mice, HIF-1α was the upstream signal of the inflammatory lung response to LCO. In vitro, the level of HIF-1α stabilization induced by LIB particles in BEAS-2B cells correlated with the intensity of lung inflammation induced by the same particles in vivo.

**Conclusions:**

We conclude that HIF-1α, stabilized in lung cells by released Co and Ni ions, is a mechanism-based biomarker of lung inflammatory responses induced by LIB particles containing Co/Ni. Documenting the Co/Ni content of LIB particles, their bioaccessibility and their capacity to stabilize HIF-1α in vitro can be used to predict the lung inflammatory potential of LIB particles.

**Electronic supplementary material:**

The online version of this article (10.1186/s12989-019-0319-z) contains supplementary material, which is available to authorized users.

## Background

Li-ion batteries (LIB) represent one of the best solutions for various electric grid applications, to improve the quality of energy harvested from wind, solar, geo-thermal and other renewable sources [1]. LIB electrodes are made of poorly water-soluble particles, micrometric in size, that might thus be respirable and biopersistent in the human respiratory tract. Exposure to LIB components is the most worrying for workers producing and handling LIB particles but future applications of LIB, such as multi-layer systems made for spray-paintable or printable DIY batteries [2–4], might extend the potential for inhalation exposure to consumers.

We previously assessed the lung toxicity of three commonly used LIB particles, lithium iron phosphate (LiFePO_4_/LFP), lithium titanium oxide (Li_4_Ti_5_O_12_/LTO) and lithium cobalt oxide (LiCoO_2_/LCO), and concluded that they represent a respiratory hazard independently of their Li content [5]. Acute inflammatory responses were recorded with the three particles. Long-term inflammation was maintained after LFP and LCO, and only LCO induced fibrotic responses. Increased hypoxia-inducible factor (HIF)-1α was recorded in the lung of mice exposed to LCO. HIF-1 is a heterodimeric transcriptional factor consisting of two subunits, HIF-1α and HIF-1β, constitutively expressed in all cells [6]. HIF-1α is the oxygen-sensitive subunit regulating the level of active HIF-1 [7]. Under normoxia, HIF-1α is continuously degraded by ubiquitin- and proteasome-dependent pathways. HIF-1α degradation is mainly controlled by the hydroxylation of two specific prolyl residues by prolyl hydroxylase. During hypoxia, HIF-1α is stabilized, heterodimerizes with HIF-1β, recruits coactivators, and induces the transcription of target genes such as interleukin (IL)-6, vascular endothelial growth factor (VEGF)-A, erythropoietin (EPO) and transforming growth factor (TGF)-β [6]. Ions such as Co^2+^ or Ni^2+^ mimic hypoxia and stabilize HIF-1α by blocking the iron-binding site of prolyl hydroxylase or directly binding to HIF-1α, thus preventing its degradation [8–10].

A wide diversity of particles, containing metals such as cobalt and/or nickel, are used in LIB electrodes [1, 11]. In view of the large variety of existing and future LIB materials, their increasing production, use and disposal, it appears essential to better identify their health hazards and to generate information about mechanisms of toxicity. Here, we investigated the role of Co and Ni and their capacity to stabilize HIF-1α in the lung responses to LIB particles*,* and evaluated the value of in vitro assays to predict their potential for lung toxicity.

## Results

### Contrasting lung toxicity of LIB particles

**Fig. 1:**
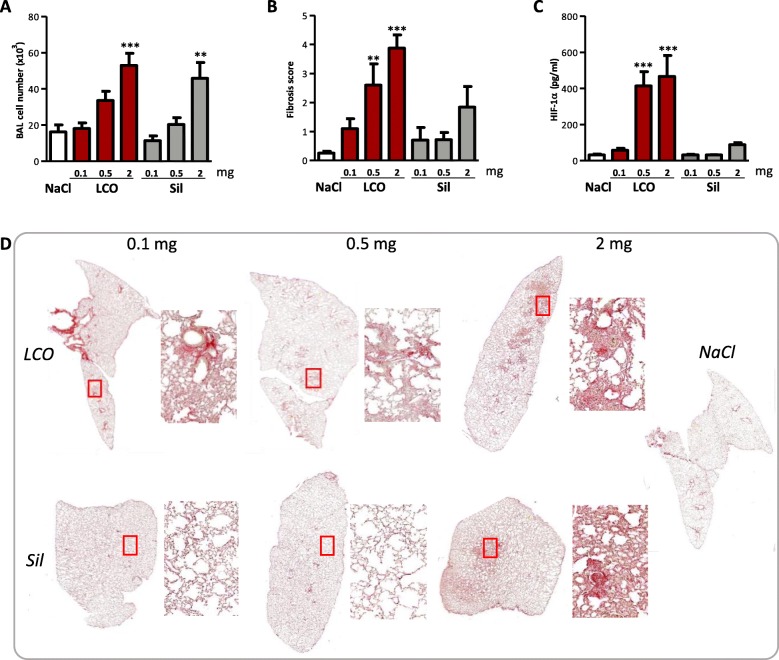
LCO induces stronger lung toxicity than silica particles. C57BL/6Jrj mice were treated with an oro-pharyngeal aspiration of NaCl (control), 0.1, 0.5 or 2 mg LCO or crystalline silica (Sil). Mice were euthanized after 2 months. Inflammatory cell infiltration (**a**) was measured in BAL; HIF-1α (**c**) in lung homogenates. Severity of fibrotic responses was scored according to Hübner et al. (2008) on lung sections stained with Sirius red (**b**). Lung sections stained with Sirius red (magnification 10x and 200x) (**d**). *P < 0.05, **P < 0.01 and ***P < 0.001 relative to NaCl-treated mice (one-way ANOVA followed by a Dunnett’s multiple comparison, *N* = 1, *n* = 5, means ± SEM)

We first documented the dose-responses for lung inflammatory and fibrotic responses induced by LCO. Mice were exposed by oro-pharyngeal aspiration to LCO (0.1, 0.5 or 2 mg). Crystalline silica particles were selected as positive control. Two months after exposure, no mortality was recorded at any of the doses tested. A clear inflammatory cell accumulation was observed from 0.5 mg LCO and at 2 mg silica (Fig. 1a). LCO also induced a stronger fibrotic response than silica (Fig. 1b, d). LCO particles are thus more potent than crystalline silica particles, despite their larger size distribution (7.21 vs > 50% fine particle fraction, respectively). HIF-1α was strongly stabilized in lung cells by LCO in a dose-dependent manner, only weakly by silica (Fig. 1c).

We next assessed the lung responses to other particles used in LIB, with different compositions (Table 1) and containing fine particles (Table 1 and Additional file 1: Figure S1). LiNiMnCoO_2_ (NMC) and LiNiCoAlO_2_ (NCA) also contain nickel. Co_3_O_4_ was used as a reference low solubility cobalt particle [12].

**Table 1:** Particle characterization

	LCO	NCA	NMC 1:1:1^a^	NMC 6:2:2	NMC 8:1:1
Density (g/cm^3^)^b^	2.37	2.95	1.93	2.84	1.93
Diameter (μm)^c^	6.47	6.13	5.16	11.34	7.53
FPF (% of total weight)^d^	7.21	2.01	1.64	1.84	2.15
Li (%)^e^	7.1	3.28	7.24	7.16	7.14
Co (%)^e^	60.2	27.86	20.22	12.12	6.03
Ni (%)^e^	/	27.75	20.22	36.35	48.28
Mn (%)^e^	/	/	18.92	11.34	5.65
Al (%)^e^	/	25.98	/	/	/
O (%)^e^	32.7	15.13	33.40	33.03	32.9

^a^The three digits reflect the Ni:Mn:Co mass ratio in the particles

^b^Measured by powder tap density

^c^Median hydrodynamic diameter determined by centrifugal liquid sedimentation (CLS) (weight-based distribution)

^d^Fine particle fraction (FPF, ≤ 5 μm) determined by Andersen cascade impaction

^e^as reported by the producers

**Fig. 2:**
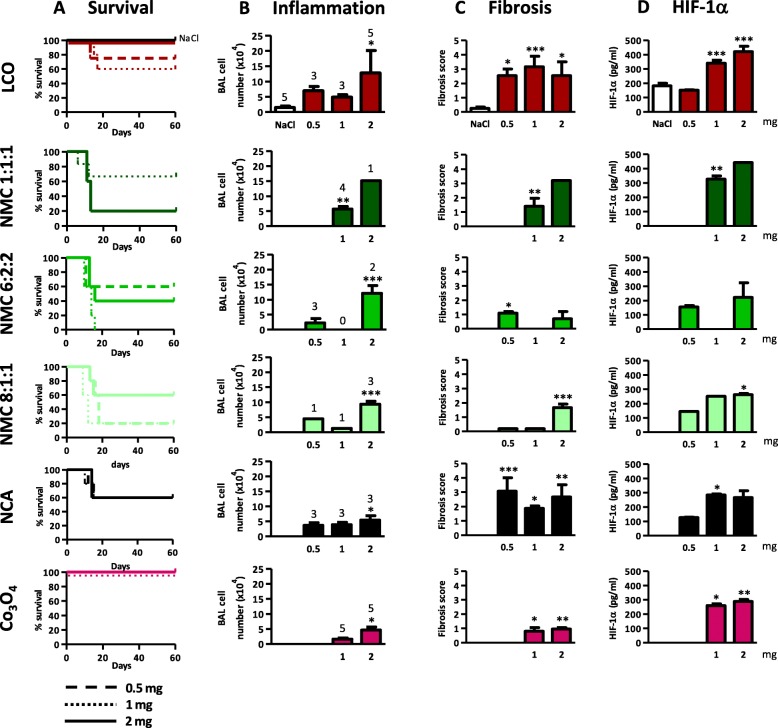
Lung inflammatory and fibrotic responses induced by LIB particles. C57BL/6Jrj mice were treated with an oro-pharyngeal aspiration of NaCl (control), 0.5, 1 or 2 mg LCO, NMC 1:1:1, NMC 6:2:2, NMC 8:1:1, NCA or Co_3_O_4_. Mortality is shown in survival curves (**a**). Surviving mice were euthanized after 2 months. Inflammatory cell infiltration (**b**) was measured in BALF. Fibrotic response (**c**) severity was scored according to Hübner et al. (2008). HIF-1α (**d**) was measured in lung homogenates. Number of surviving mice is indicated for each condition above the columns (**b**). *P < 0.05, **P < 0.01 and ***P < 0.001 relative to NaCl-treated mice (one-way ANOVA followed by a Dunnett’s multiple comparison or t test, *N* = 1, *n* = 4–6 (treated mice), means ± SEM)

Mice were first exposed to 1 or 2 mg LIB particles by oro-pharyngeal aspiration. Surprisingly, mice died shortly after exposure, except with Co_3_O_4_ (Fig. 2a). The dose was then reduced to 0.5 mg for the most active particles (NMC6:2:2, NMC8:1:1, NCA and LCO). After 2 m, all LIB particles induced inflammation (cell infiltration) at the dose of 2 mg (Fig. 2b). Co_3_O_4_ induced a slight inflammation. Lung fibrotic manifestations (score ≥ 2) were induced by LCO and NCA particles at 0.5 mg whereas, at 1 and 2 mg, LCO, NMC1:1:1 and NCA induced clear fibrotic changes with fibrotic masses (Fig. 2c). NMC 6:2:2, 8:1:1 and Co_3_O_4_ induced some isolated fibrotic changes (See Additional file 1: Figure S2). All particles induced different (dose-dependent) levels of HIF-1α stabilization (Fig. 2d). As these LIB particles induced acute toxicity (mortality) and severe lung responses, it appeared crucial to identify the mechanisms of this toxicity.

**Fig. 3:**
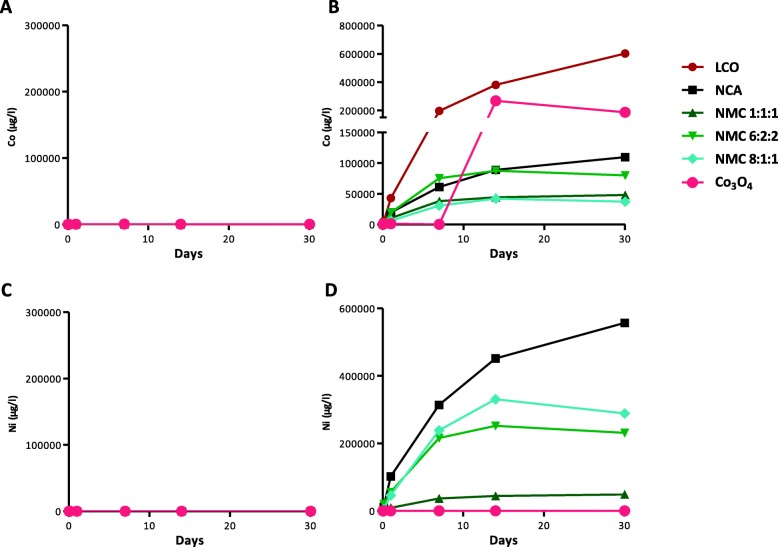
Bioaccessibility of Co and Ni from LIB particles. LIB particles and Co_3_O_4_ were incubated in artificial fluids mimicking the extracellular (pH 7.3) (**a**, **c**) or the phagolysosomal (pH 4.2) (**b**, **d**) compartment. Particles were incubated at 37 °C under gentle agitation and released Co (**a**, **b**) and Ni concentrations (**c**, **d**) were determined by ICP-MS in the SN after centrifugation of an aliquot of the suspensions after 3, 24 h, 7, 14 and 30 days

We first focused on the determinants of acute toxicity. Because tested LIB particles contained Co and/or Ni, 2 elements able to induce pulmonary toxicity [13–15], we assessed the bioaccessibility of these metals in artificial fluids mimicking the extracellular (pH 7.3) and the phagolysosomal (pH 4.2) cellular compartments over a period of 30 days (Fig. 3). The amount of Co released at pH 7.3 was very low for all particles (Fig. 3 a) in comparison to pH 4.2 (Fig. 3b), suggesting that Co ions can be released in the phagolysosomes. The pattern of Co release at pH 4.2 was different for all LIB particles, LCO releasing the largest amount of Co. Co_3_O_4_ particles released more Co than the other LIB particles. NCA and NMC6:2:2 released a higher amount of Co than NNC1:1:1 and NMC8:1:1. At pH 7.3, Ni bioaccessibility was also very low (Fig. 3c). At pH 4.2, the Ni bioaccessibility pattern was different for all LIB particles, NCA and NMC8:1:1 releasing the highest amount of Ni (Fig. 3d). Thus, the patterns of Co and Ni released from LIB particles did not follow their Co or Ni % content. We next performed a bivariate analysis to identify the determinants of acute toxicity (mortality) after exposure to LIB particles. We analyzed the implication of the Co and Ni amount contained in the administered doses (0.5, 1 or 2 mg LIB particles) and their bioaccessibility (calculated from ion % released at pH 4.2) either separately or together (Table 2). The acute toxic potential (mortality) of these LIB particles was related to the Ni content and its release from the particles.

**Table 2:** Correlation between mortality recorded at day 18 and Ni/Co content or Ni/Co released by the particles

	Correlation coefficient	*p*-value
*Co content*	−0.064	0.801
***Ni content***	**0.760**	**0.0002**
*Bioaccessible Co*	−0.079	0.755
***Bioaccessible Ni***	**0.719**	**0.001**
*Ni + Co content*	0.163	0.517
*Bioaccessible Ni + Co*	0.176	0.484

Mice were treated with 0.5, 1 or 2 mg LIB particles (see Fig. 2). Bivariate analysis (Spearman Rho) between the mortality % at day 18, and the particle Ni and/or Co content (μg) or the amount of Ni and/or Co ions (μg) released. Co and/or Ni contents were calculated from the administered doses and from the Co and/or Ni % of the particles. Bioaccessible Co and/or Ni represent the amount of bioaccessible ions, calculated from the Co/Ni contained in the administered doses and the % released at pH 4.2 at day 14 (mortality was recorded between day 6 and 18). Significant relationships are identified in bold

### HIF-1α is a determinant of the lung inflammation induced by LIB particles

We next performed similar analyses to identify the determinants of the late inflammatory response (inflammatory cell infiltration in the broncho-alveolar lavage (BAL) 2 months after administration) (Table 3), including lung HIF-1α content, BAL fuid (BALF) lactate deshydrogenase (LDH) activity as a marker of cytotoxicity, Ni and/or Co contents of LIB particles and their bioaccessibility (calculated from the % released at pH 4.2). HIF-1α stabilization in lung tissue, the sum of Co and Ni content in particles and their summed bioaccessibility significantly correlated with lung inflammation (Table 3). The sum of Ni and Co content and their bioaccessibility also positively correlated with the stabilization of HIF-1α (*r* = 0.539, *p*-value = 0.0001 and *r* = 0.500, *p*-value = 0.0001 respectively). The same analysis did not show any significant association of the same variables with fibrotic responses (data not shown). These results thus supported the hypothesis that HIF-1α stabilization induced by Co and Ni ions drives the lung inflammatory responses of these LIB particles.

**Table 3:** Tracing the determinants of lung inflammation induced by LIB particles after 2 months

	Correlation coefficient	*p*-value
*LDH*	0.183	0.195
***HIF-1α***	**0.311**	**0.025**
*Co content*	0.245	0.080
*Ni content*	0.065	0.270
*Bioaccessible Co*	−0.020	0.886
*Bioaccessible Ni*	0.035	0.806
***Ni + Co content***	**0.480**	**0.0003**
***Bioaccessible Ni + Co***	**0.445**	**0.0004**

Bivariate analysis (Pearson correlation) between the inflammatory cell recruitment, selected as marker of lung inflammation and HIF-1α, LDH activity, the particle Ni and/or Co content (μg) or the amount of Ni and/or Co ions (μg) released. Inflammatory cell recruitment was assessed in the BAL, HIF-1α (μg/ml) was measured in lung homogenates, LDH activity (iU/l) in BALF. Co and/or Ni contents were calculated from the administered doses and from the Co and/or Ni % of the particles. Bioaccessible Co and/or Ni represent the amount of bioaccessible ions, calculated from the Co/Ni contained in the administered doses and the % released at pH 4.2 at day 30. Significant relationships are identified in bold

### HIF-1α mediates lung inflammation induced by LCO

**Fig. 4:**
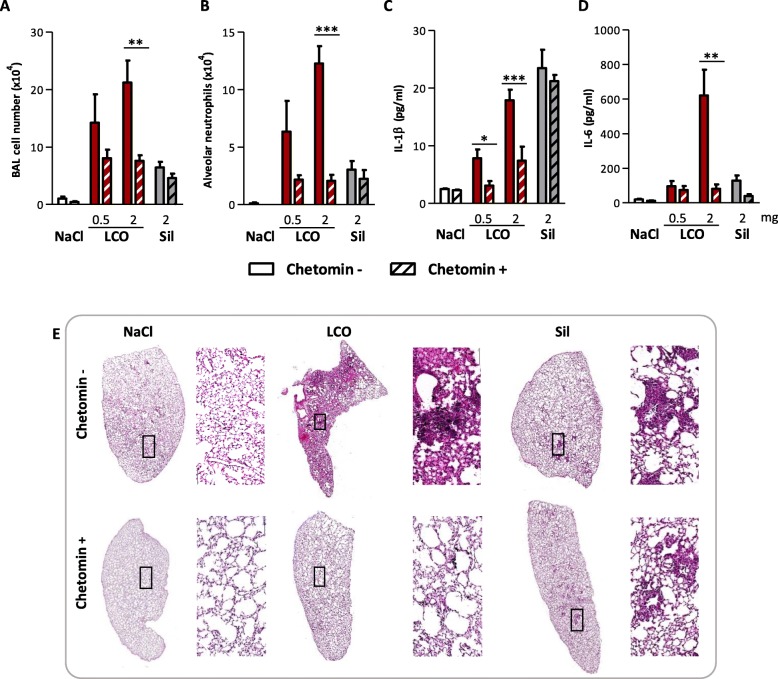
HIF-1α drives lung inflammation induced by LCO. C57BL/6Jrj mice were treated with an oro-pharyngeal aspiration of NaCl (control), 0.5 or 2 mg LCO or crystalline silica (Sil). Mice received i.p. injections of 0.5 mg/kg bw/d chetomin or vehicle (saline solution with 10% DMSO) at day − 1, 1 and 2 and were enthanized at day 3. Alveolar inflammatory cell infiltration and neutrophils were assessed in the BAL (**a**, **b**). IL-1β (**c**) and IL-6 (**d**) were measured in BALF; Lung sections of mice exposed to 2 mg particles were stained with HE (magnification 10x and 200x) (**e**). *P < 0.05, **P < 0.01 and ***P < 0.001 (t-test between chetomin - and + mice for each condition, *N* = 1, *n* = 5, means ± SEM)

We next evaluated the implication of HIF-1α in the toxic activity of LIB particles by inhibiting its activity with chetomin, a disrupter of HIF binding to its transcriptional co-activator P300 [16]. This was assessed with LCO, the LIB particle inducing the strongest lung inflammation and fibrotic responses, and crystalline silica particles as control. We previously observed that LCO early stabilized HIF-1α in the lung 3 days after exposure and later after 2 months. Crystalline silica weakly stabilized HIF-1α only 2 months after exposure (Fig. 1), suggesting that late inflammatory of fibrotic responses might also contribute to HIF-1α stabilization [5]. Therefore, we first focused on the implication of HIF-1α in early lung responses to isolate the specific effect of LCO. Three days after administration of 0.5 or 2 mg LCO, inflammatory cell recruitment including neutrophils and the pro-inflammatory cytokines IL-1β and IL-6 in BALF were largely reduced by the inhibition of HIF-1α (Fig. 4a, b, c, d). This reduction was not observed in silica-exposed mice. To further assess inflammation, lung sections of mice exposed to 2 mg particles were stained with hematoxylin and eosin (HE). Aggregates of lymphocytes and macrophages, and accumulation of cellular debris were observed in LCO lungs to a larger extent than in silica lungs (Fig. 4e). In LCO lungs from mice treated with the inhibitor, inflammation was drastically reduced. In silica lungs, the formation of inflammatory foci was not prevented by chetomin (Fig. 4e). These results suggested that HIF-1α is specifically implicated in early lung inflammation induced by LCO. To confirm this observation, we used PX-478, another inhibitor of HIF-1α responses targeting a different pathway. PX-478 inhibits HIF-1α by decreasing its translation and transcription, as well as de-ubiquitination [17–19]. Similar results were observed with this inhibitor (See Additional file 1: Figure S3), confirming the implication of HIF-1α in early lung inflammation induced by LCO. Similar experiments were conducted over a period of 1 and 2 months to assess the potential implication of HIF-1α in later lung responses to LCO, but mice did not tolerate a longer treatment with chetomin or PX-478 (not shown).

### HIF-1α stabilization acts upstream of lung inflammation

**Fig. 5:**
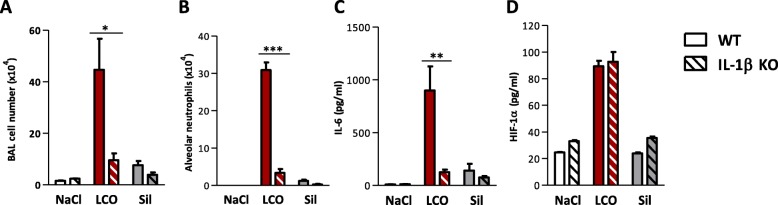
HIF-1α stabilization after LCO acts upstream of inflammation. C57BL/6Jrj or IL-1β KO mice were treated with an oro-pharyngeal aspiration of NaCl (control), 2 mg LCO or crystalline silica (Sil). Mice were euthanized after 3 days. Alveolar inflammatory cell infiltration (**a**) and neutrophils (**b**) were assessed in the BAL, and IL-6 (**c**) was measured in BALF; HIF-1α in lung homogenates (**d**). *P < 0.05, **P < 0.01 and ***P < 0.001 (t-test between WT and IL-1β KO mice for each condition, *N* = 1, *n* = 5, means ± SEM)

We next investigated the possible influence of lung inflammation on HIF-1α stabilization. To minimize the number of animals used, IL-1β knock-out (KO) mice were treated only with 2 mg of LCO or crystalline silica particles and compared to their wild-type (WT) counterparts (C57BL/6JRj) after 3 days. Inflammatory cells, including neutrophils, were recruited after LCO and crystalline silica particles in WT mice. Only LCO induced HIF-1α stabilization in WT mice. All inflammatory parameters induced in WT mice were strongly reduced in IL-1β KO mice after LCO and (although not significantly) after silica (Fig. 5a, b, c). HIF-1α stabilization induced by LCO was, however, not modified in IL-1β KO mice (Fig. 5d), indicating that HIF-1α stabilization induced by LCO particles is not a consequence of inflammation.

### In vitro HIF-1α stabilization predicts the lung inflammatory potential of LIB particles

**Fig. 6:**
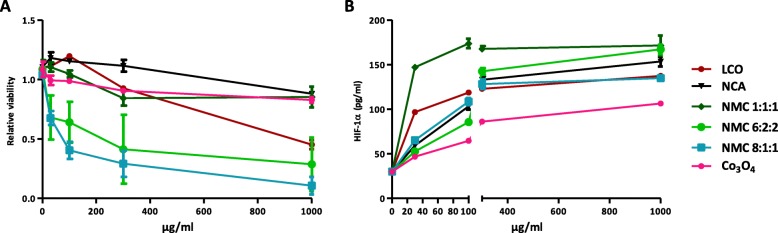
Cytotoxicity and HIF-1α stabilization induced by LIB particles in BEAS-2B cells. BEAS-2B cells were exposed to NaCl (control), 30, 100, 300 or 1000 μg/ml of LCO, NCA, NMC 1:1:1, NMC 6:2:2, NMC 8:1:1 or Co_3_O_4_. Cytotoxicity was assessed after 24 h (**a**) by the WST-1 assay. HIF-1α (**b**) protein contents were measured in cell lysates. (*N* = 2, *n* = 4, means ± SEM)

**Fig. 7:**
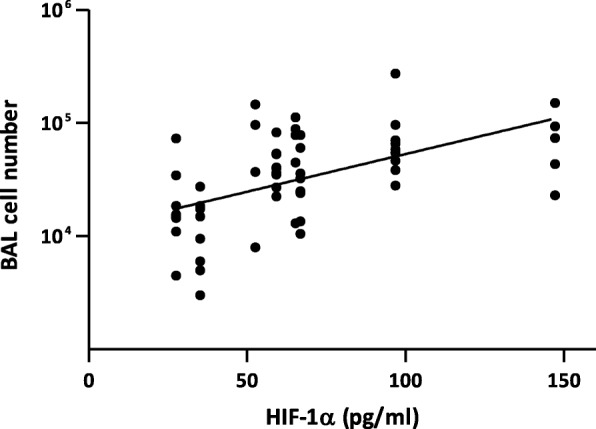
Correlation between inflammation induced by LIB particles in vivo and HIF-1α stabilization in vitro. Scatter graph and bivariate analysis demonstrate a positive correlation between the inflammatory cell recruitment level induced by LIB particles after 2 months in mice (see Fig. 2) and the stabilization of HIF-1α (pg/ml) in BEAS-2B at the concentration of 30 μg LIB particle/ml (see Fig. 6). (y = 0.006671 x + 4.06; *p* < 0.0001; *r* = 0.532)

To further substantiate the mechanistic association between HIF-1α stabilized by Co/Ni ions and lung inflammation (Table 3), we next assessed in vitro the HIF-1α response to LIB particles in BEAS-2B cells. This simplified model allows isolating the HIF-1α response from the multiple inflammatory components present in the lung. BEAS-2B cells were exposed to increasing doses of LIB particles (30 to 1000 μg/ml), the lowest concentration matching in vivo doses (See Additional file 1: Figure S4). Higher concentrations were also tested because, in vivo*,* lung cells were exposed to Co ions released during 2 months while, in vitro, cells were exposed only during 24 h. NMC8:1:1 and 6:2:2 were cytotoxic at 30 μg/ml and LCO at 1000 μg/ml. A very slight cytotoxicity appeared at 1000 μg/ml for the other particles (Fig. 6a). At 30 and 100 μg/ml, HIF-1α stabilization was stronger for LCO and NMC1:1:1. At 1000 μg/ml, all LIB particles induced a strong HIF-1α stabilization (Fig. 6b). Co_3_O_4_, which did not induce high lung inflammation in vivo compared to the LIB particles (Fig. 2), induced a weaker HIF-1α stabilization than LIB particles in vitro. Using the results of in vivo experiments (Fig. 2), bivariate analyses revealed a positive correlation between the in vivo inflammatory response (BAL inflammatory cell infiltration) and in vitro HIF-1α stabilization in BEAS-2B cells. Analyses were performed on HIF-1α stabilization at all doses tested in vitro (30, 100, 300 or 1000 μg particles/ml). All correlations were significant, the relation at 30 μg particles/ml which best matches the in vivo doses is illustrated here (Fig. 7). These results confirm that the simple presence and release of Co/Ni from LIB particles and their ability to stabilize HIF-1α determine the in vivo inflammation, independently of all other possible components.

Compared to % Co + Ni content or bioaccessibility, HIF-1α stabilization in vitro integrates a number of factors such as intracellular distribution of Co/Ni ions, and measures the bioactivity of these elements, offering an additional option to predict the toxic potential of (new) LIB particles, hence reducing in vivo testing. The measurement of HIF-1α stabilization in vitro in BEAS-2B cells, the Co/Ni content of LIB particles and their bioaccessibility are, therefore, useful predictors of the lung inflammatory potential of LIB particles.

## Discussion

We observed here that LIB particles containing Co and/or Ni induce lung inflammation and even fibrotic responses in mice. We show that Co and/or Ni contents and bioaccessibility, as well their capacity to stabilize HIF-1α, are determinants of lung inflammation. We also demonstrated that HIF-1α in lung cells mediates LCO-induced inflammation and is an upstream signal of the responses. In addition to the well-known implication of HIF in the development of cancer, invasion and metastasis, HIF-1α also plays many roles in inflammation, induces the secretion of inflammatory mediators and promotes myofibroblast differentiation as well as epithelial-mesenchymal transition via the TGF-β pathway [6, 20–22].

Pulmonary diseases including cancer, asthma and fibrosing alveolitis have previously been reported in workers exposed to cobalt [13, 14]. Cobalt compounds can induce cytotoxicity, apoptosis, inflammatory responses and genotoxicity in vitro [23]. Some of the effects of cobalt are related to its high affinity for sulfhydryl groups leading to enzyme inactivation, antagonism for Ca^2+^ channel cell signalling, production of reactive oxygen species leading to oxidative stress, and finally to its ability to stabilize HIF-1α [23, 24]. LCO containing the highest % of Co among the range of particles tested, and Co being suspected to be the element responsible for the high toxicity of LCO, we hypothesized that others particles (with lower Co content) would induce lower lung effects than LCO. However, acute toxicity was observed early after exposure. In addition to cobalt, some LIB particles contain nickel, which appears as the element responsible for the observed mortality. Previous studies have shown that acute inhalation exposure to Ni induced lethal injury characterized by inflammatory cell infiltration, haemorrhage and destruction of alveolar epithelial cells [25, 26]. Chronic exposure to Ni can lead to asthma, inflammation, pulmonary fibrosis, kidney diseases and cancer [10, 15]. Like Co, Ni is able to stabilize HIF-1α by blocking the iron-binding group of the prolyl hydroxylase [10]. As inhibition of prolyl hydroxylase activates nuclear factor (NF)-κB [27], Co and Ni ions can thus both participate to the activation of NF-κB, leading to inflammation, as previously suggested [28]. The Co and Ni contents of LIB particles and their bioaccessibility are, indeed, correlated to the in vivo lung inflammation in the present study.

HIF-1α was also correlated to the in vivo lung inflammation induced by LIB particles. Moreover, inhibition of HIF-1α led to a reduction of lung inflammation induced by LCO particles, indicating the key pathogenic role of this transcription factor. HIF-1α can promote NF-κB activity in macrophages, neutrophils and non-immune cells, resulting in the transcription of target genes of inflammation such as pro-inflammatory cytokines (tumor necrosis factor (TNF)-α, IL-6, IL-1, IL-12) [29, 30]. To test the implication of HIF-1α in LCO lung inflammation, we first used chetomin which binds to the Zn^2+^-binding cysteine/histidine rich 1 (CH1) domain of p300, leading to a reduction of the interaction between HIF-1α and P300 [16] and reduces the expression of HIF-1α target genes [16, 31]. Chetomin drastically reduced LCO lung inflammation. These observations were confirmed by the use of PX-478 which inhibits HIF-1α transcription and translation by another mechanism than chetomin [16, 18]. Thus, we showed here, for the first time, the implication of HIF-1α in lung inflammation induced by particles containing Co and/or Ni. We conclude that inflammation induced by LCO is dependent on HIF-1α.

In addition, the HIF-1α response to LCO was maintained in the absence of inflammation in IL-1β KO mice. We can thus conclude that HIF-1α is an upstream signal of the lung inflammatory responses to LIB particles containing Co. These results are consistent with Rius et al. [32] who showed that if NF-κB can regulate HIF-1α transcription in activated macrophages, NF-κB activation alone is, however, not sufficient to stabilize HIF-1α, indicating that both transcriptional and post-transcriptional (like Co and Ni ions) regulators are implicated in HIF-1α production/stabilization.

The Co and Ni content of LIB particles and their bioaccessibility at pH 4.2 represent thus good indicators of the toxic potential of LIB particles. To refine the predictive information, we can also measure HIF-1α protein stabilization in BEAS-2B cells exposed to LIB particles. A study comparing 10 commonly used cell lines concluded that BEAS-2B cells are useful for toxicological studies because they exhibit the highest homology in gene expression pattern with human primary cells and the lowest number of dysregulated genes compared with non tumoral lung tissues [33]. Moreover, BEAS-2B have been previously used to study the toxicity of cobalt compounds [12, 34]. The BEAS-2B cell line is thus an appropriate model to evaluate the lung inflammatory potential of LIB particles by measuring the stabilization of the key mediator HIF-1α.

A large variety of materials are in use in or under consideration for the development of LIB materials. While micro-sized particles, as tested in the present study, are currently used in most commercialized batteries, nano-sized materials are in intense development to improve technical performances [35]. The nanosize is a plausible source of additional concern as it can result in more severe hazardous properties and increased particle exposure via inhalation. Thus, toxicological evaluation of existing and newly developed LIB particles appears as a priority. We identified the Ni and Co content of LIB particles and their bioaccessibility, as well as HIF-1α as key determinants of the lung inflammatory responses to LIB particles. Evaluating HIF-1α levels in BEAS-2B cells exposed to LIB particles is a predictor of their inflammatory potential.

## Conclusions

We report the implication of HIF-1α induced by Ni and Co ions in lung inflammatory responses induced by LIB particles. HIF-1α is the upstream signal of the inflammatory responses induced by these LIB particles, participating to the secretion of IL-1β. Documenting the amount of Co and Ni in LIB particles, their bioaccessibility as well as HIF-1α stabilization in BEAS-2B cells, predict the lung toxicity of LIB particles.

## Methods

### Particles

LCO (LiCoO_2_) was obtained from MTI Corporation (Richmond, USA), NCA (LiNiCoAlO_2_), NMC 1:1:1 (LiNi_0.33_Co_0.33_Mn_0.33_O_2_), NMC 6:2:2 (LiNi_0.6_Co_0.2_Mn_0.2_O_2_) and NMC 8:1:1 (LiNi_0.8_Co_0.1_Mn_0.1_O_2_) from Umicore, cobalt oxide (Co_3_O_4_, size < 10 μm, 221,643) from Sigma-Aldrich (Missouri, USA) and micrometric crystalline silica particles (Min-U-Sil 5, d_50_ 1.6 μm) from US Silica (Berkley Springs, USA). Before all experiments, particles were heated 2 h at 200 °C to inactivate any possible endotoxin or other microbial contaminants.

### Particle solubilization

To assess the bioaccessibility of elements contained in LIB particles, 10 mg particles were incubated in 10 ml artificial fluids mimicking the extracellular (pH 7.3) or the phagolysosomal (pH 4.2) compartments as previously described [36]. Particles were incubated during 30 days at 37 °C under gentle agitation. One ml aliquots were collected after 3, 24 h, 7, 14 and 30 days and centrifuged (20,000 *g*, 10 min). Element concentrations were determined in the SN by ICP-MS.

### Particle characterization

The density of LIB particles was assessed by tap density measurement and their aerodynamic size distribution in an Andersen cascade impactor (1 ACFM Eight Stage Non-Viable Cascade Impactor, Graseby Andersen, Atlanta, USA) as previously described [5]. The particle size distribution, based on the hydrodynamic diameter was also assessed by CLS on a DC24000 system (CPS instruments Inc., Stuart, Florida, USA), equipped with a 405-nm wavelength laser detector, with PVC standard (nominal particle size = 719 nm). Sizes are expressed in terms of hydrodynamic diameter assuming all particles are spherical. Each measurement was done by injecting 0.1 ml of a 1 mg particle/mL suspension in NaCl 0.9%.

### Animals and treatments

Female C57BL/6JRj mice were purchased from Janvier Labs (St Bertevin, France). Interleukin (IL)-1β deficient (knock-out, ^−/−^) mice (C57BL/6 J background) were obtained from the Transgenose Institute (Orleans, France). Eight-week-old animals were kept with sterile rodent feed and acidified water, and housed in positive-pressure air-conditioned units (25 °C, 50% relative humidity) on a 12 h light/dark cycle. Particles were suspended in sterile 0.9% saline. Mice were randomly allocated to experimental groups. After anaesthesia with a mix of Nimatek, 1 mg/mouse (Eurovet, Bladel, Nederland) and Rompun, 0.2 mg/mouse (Bayer, Kiel, Germany) given intraperitoneally, a 50 μl suspension of particles or NaCl (controls) was directly administered by oro-pharyngeal aspiration. Single dose administration of particles is a convenient alternative to inhalation exposure for initial hazard identification [37, 38] and induces qualitatively similar lung responses as inhalation exposure [39, 40]. Crystalline silica particles were used as reference material. Inflammatory and fibrotic responses are recorded in mice with a dose of 2 mg crystalline silica particles administered via oro-pharyngeal aspiration [41–44]. LIB particles were tested at doses of 0.1, 0.5 or 2 mg to allow benchmarking of their respiratory toxicity relative to crystalline silica particles.

Chetomin (Sigma-Aldrich) or vehicle (saline solution with 10% dimethylsulfoxide (DMSO, Sigma-Aldrich)) was injected intraperitoneally at 0.5 mg/kg bw/d for the 3 days experiment, one day before administration of the particles and during the 2 following days, or 3 times per week for long term experiments. PX-478 (S-2-amino-3-[4′-N,N,-bis (2-chloroethyl)amino] phenyl propionic acid N-oxide dihydrochloride, Cayman Chemicals, Michigan, USA) or vehicle (saline solution with 10% DMSO) was injected intraperitoneally at 20 mg/kg bw/d, one day before administration of the particles and during the 2 following days. Mice were euthanized 3 days or 2 months after particle administration with an intraperitoneal injection of 12 mg sodium pentobarbital (Certa, Braine-l’Alleud, Belgium).

### Broncho-alveolar lavage and lung sampling

Broncho-alveolar lavage (BAL) was performed by cannulating the trachea and infusing the lungs with 1 ml NaCl 0.9%. Whole lungs were then perfused with NaCl 0.9% and excised. Left lobes were placed in 3.65% paraformaldehyde (Sigma-Aldrich, St Louis, Missouri, USA) in phosphate buffered saline (PBS) for later histological analysis, and remaining lobes in liquid nitrogen or lysis buffer for homogenization. Lungs were homogenized on ice with an Ultra-Turrax T25 (Janke and Kunkel, Brussels, Belgium) and stored at − 80 °C. BAL were centrifuged 10 min at 4 °C (240 *g*). Cell-free supernatant (BALF) was used for biochemical measurements. After resuspension of the pellet in PBS, total BAL cells were counted in Turch (crystal violet 1%, acetic acid 3%) and cytocentrifuged for differentiation by light microscopy after Diff-Quick staining (200 cells counted, Polysciences, Warrington, UK). Lactate dehydrogenase (LDH) activity was assayed on BALF as described previously [42].

### Quantification of IL-1β, IL-6 and HIF-1α

IL-1β and IL-6 were quantified by enzyme-linked immunosorbent assay (ELISA) (Limit of detection (LOD): 7.8 pg/ml, DuoSet ELISA, R&D Systems, Minneapolis, USA) in BALF following manufacturer’s instructions. HIF-1α (LOD: 31.25 pg/ml, DuoSet ELISA, R&D Systems) was assessed in SN of lung homogenates (centrifuged 10 min at 240 *g*, 4 °C) or in the cell culture after lysis following manufacturer’s instructions.

### Histology and fibrosis scoring

Paraffin-embedded lung sections were stained with HE (lung structure staining) or Sirius Red (type I and III collagen staining). The sections were scanned (Leica SCN400, Brussels, Belgium) and examined with Tissue Image Analysis 2.0 (Leica Biosystems). Fibrotic responses was quantified using a modified Ashcroft scale (grade 0 to 8) standardized for fibrosis evaluation in small animals (clear fibrotic changes are observed from the grade 2) [45]. Analyses were performed under blind conditions by the same investigator.

### Cell culture and in vitro exposure

BEAS-2B cells (human bronchial epithelial cell line, ATCC, Virginia, USA) were cultured at 37 °C in complete medium, i.e. LHC-9 medium (Gibco, Paisley, UK) supplemented with 1% antibiotic-antimycotic (Gibco) on coated surfaces. Culture flasks and plates were precoated with a mixture (60 μl/cm^2^) of 0.01 mg/mL fibronectin (Fibronectin from human plasma 0.1%, Sigma), 0.03 mg/mL bovine collagen type I (collagen coating solution 50 μg/ml, Sigma) and 0.01 mg/mL bovine serum albumin (7.5% in PBS, Sigma) at least 2 h at 37 °C and then washed 1 x with PBS (Gibco, Paisley, UK) before cell seeding. Cells were subcultured and exposed before reaching confluence. Before exposure, BEAS-2B were plated in 96-well plates or 48-well plates (30 000 cells/cm^2^ culture well surface area) in complete medium. After 24 h, cells were exposed to the particles during 24 h in culture medium (150 μl/well for 48-well plates (0.95 cm^2^/w) and 100 μl/well for 96-well plates (0.32 cm^2^/w)). Given the similar size and density of the tested particles (Table 1), differential sedimentation and cellular doses are unlikely to confound the results. All tested particles directly sedimented in the cell culture well.

Supernatants (SN) of cell culture were collected and stored at − 80 °C for later analysis. Cells were then washed once with LHC basal medium and viability was evaluated by using the water soluble tetrazolium salts (WST-1) assay (Roche, Mannheim, Germany, 5%) following manufacturer’s instructions (96-well plates). Cells cultured in 48-well plates were washed and lysed for HIF-1α dosage following manufacturer’s instructions.

### Statistics

Graphs and statistical analyses were performed with GraphPad Prism 5.0 and/or Microsoft excel 2013. Bivariate analyses were performed with IBM SPSS statistics 25. All results are expressed as means ± standard errors on the mean (SEM). Differences between control and treated groups were evaluated by one-way analysis of variance (ANOVA), followed by a Dunnett’s multiple comparison, or by a t test. Statistical significance was considered at *P* < 0.05.

## Additional file

Additional file 1: **Figure S1.** Particle size distributions. LCO (a, d), NCA (b, e), NMC 1:1:1 (c, f), NMC 6:2:2 (g, i) and NMC 8:1:1 (h, j) size distributions (weight based distributions (a-c, g-h) and number based distributions (d-f, i-j)) assessed by centrifugal liquid sedimentation. **Figure S2.** Lung sections of mice 2 months after treatment with LIB particles. C57BL/6Jrj mice were treated with an oro-pharyngeal aspiration of NaCl (control), 0.5, 1 or 2 mg LCO, NMC 1:1:1, NMC 6:2:2, NMC 8:1:1, NCA or Co_3_O_4_. Lung sections were stained with Sirius red (magnification 200x). **Figure S3.** HIF-1α drives lung inflammation induced by LCO. C57BL/6Jrj mice were treated with an oro-pharyngeal aspiration of NaCl (control) or 2 mg LCO. Mice were treated with i.p. injections of 20 mg/kg bw/d PX-478 or with the vehicle (saline solution with 10% DMSO) at day − 1, 1 and 2. Mice were euthanized after 3 days. Inflammatory cell infiltration was assessed in the BAL (a). Lung sections were stained with HE (magnification 10x) (b). *P < 0.05, **P < 0.01 and ***P < 0.001 (t-test between PX-478 - and + mice for each condition, *N* = 1, *n* = 5, means ± SEM). **Figure S4.** Comparison of in vivo and in vitro doses. (PDF 1102 kb)

## Data Availability

The datasets used and/or analyzed during the current study are available from the corresponding author on reasonable request.

## Abbreviations

BAL: Broncho-alveolar lavage
BALF: BAL fluid
CH1: Zn^2+^-binding cysteine/histidine rich 1
CLS: Centrifugal liquid sedimentation
DMSO: Dimethylsulfoxide
ELISA: Enzyme-linked immunosorbent assay
FPF: Fine particle fraction
HE: Hematoxylin and eosin
HIF: Hypoxia-inducible factor
ICP-MS: Inductively coupled plasma mass spectrometry
IL: Interleukin
LCO: LiCoO_2_
LDH: Lactate dehydrogenase
LFP: LiFePO_4_
LIB: Lithium-ion battery
LOD: Limit of detection
LTO: Li_4_Ti_5_O_12_
NCA: LiNiCoAlO_2_
NMC: LiNiCoMnO_2_
PBS: Phosphate buffered saline
PX-478: S-2-amino-3-[4′-N,N,-bis (2-chloroethyl)amino] phenyl propionic acid N-oxide dihydrochloride
SEM: Standard error on the mean
SN: Supernatant
TGF: Transforming growth factor
TNF: Tumor necrosis factor
TWA: Time weighted average
WST-1: Water soluble tetrazolium salts-1
WT: Wild-type
ρ: Density
