# Supplementary material for: HIF-1α is a key mediator of the lung inflammatory potential of lithium-ion battery particles
Source: Part Fibre Toxicol. 2019 Sep 18;16:35. doi: 10.1186/s12989-019-0319-z (PMC6751682; doi:10.1186/s12989-019-0319-z)
Supplement: Supplementary file 1 — Figure S1. Particle size distributions. LCO (a, d), NCA (b, e), NMC 1:1:1 (c, f), NMC 6:2:2 (g, i) and NMC 8:1:1 (h, j) size distributions (weight based distributions (a-c, g-h) and number based distributions (d-f, i-j)) assessed by centrifugal liquid sedimentation. Figure S2. Lung sections of mice 2 months after treatment with LIB particles. C57BL/6Jrj mice were treated with an oro-pharyngeal aspiration of NaCl (control), 0.5, 1 or 2 mg LCO, NMC 1:1:1, NMC 6:2:2, NMC 8:1:1, NCA or Co3O4. Lung sections were stained with Sirius red (magnification 200x). Figure S3. HIF-1α drives lung inflammation induced by LCO. C57BL/6Jrj mice were treated with an oro-pharyngeal aspiration of NaCl (control) or 2 mg LCO. Mice were treated with i.p. injections of 20 mg/kg bw/d PX-478 or with the vehicle (saline solution with 10% DMSO) at day − 1, 1 and 2. Mice were euthanized after 3 days. Inflammatory cell infiltration was assessed in the BAL (a). Lung sections were stained with HE (magnification 10x) (b). *P < 0.05, **P < 0.01 and ***P < 0.001 (t-test between PX-478 - and + mice for each condition, N = 1, n = 5, means ± SEM). Figure S4. Comparison of in vivo and in vitro doses. (PDF 1102 kb) [file 12989_2019_319_MOESM1_ESM.pdf]

Particle and Fibre Toxicology

Additional file 1

HIF-1α is a key mediator of the lung inflammatory potential of lithium-ion battery particles

Violaine Sironval[§], Mihaly Palmai-Pallag, Rita Vanbever, François Huaux, Jorge Mejia, Stéphane Lucas, Dominique Lison, Sybille van den Brule

[§]Corresponding author information:
Email address: violaine.sironval@uclouvain.be
Affiliation: Louvain centre for Toxicology and Applied Pharmacology, Institut de Recherche Expérimentale et Clinique, UCLouvain

Table of contents

Figure S1: Particle size distributions

Figure S2: Lung sections in surviving mice 2 months after treatment with LIB particles

Figure S3: HIF-1α drives lung inflammation induced by LCO

Figure S4: Comparison of *in vivo* and *in vitro* doses

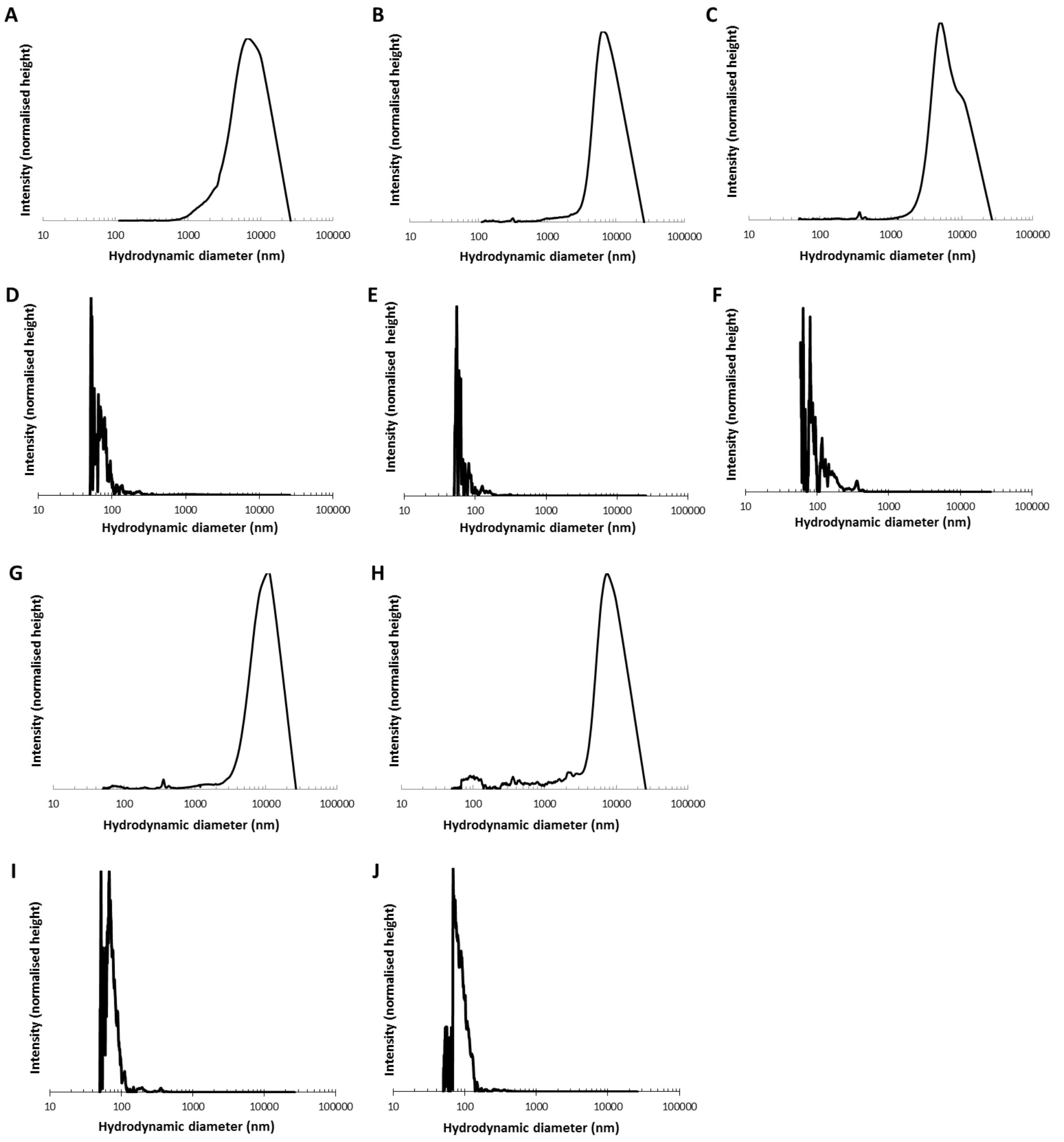

Figure S1: Particle size distributions. LCO (a, d), NCA (b, e), NMC 1:1:1 (c, f), NMC 6:2:2 (g, i) and NMC 8:1:1 (h, j) size distributions (weight-based distributions (a-c, g-h) and number-based distributions (d-f, i-j)) assessed by centrifugal liquid sedimentation.

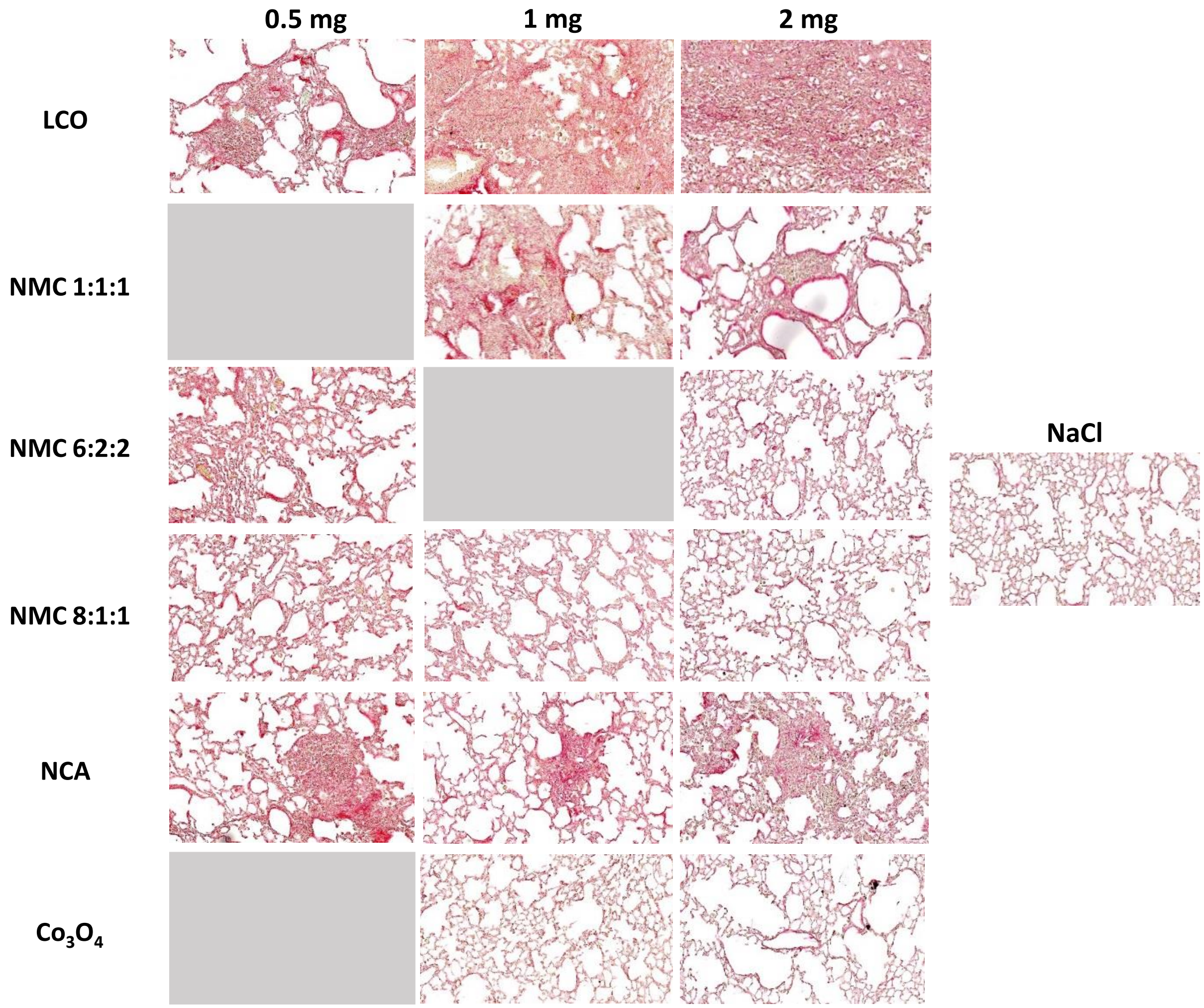

Figure S2: Lung sections in surviving mice 2 months after treatment with LIB particles. C57BL/6Jrj mice were treated with an oro-pharyngeal aspiration of NaCl (control), 0.5, 1 or 2 mg LCO, NMC 1:1:1, NMC 6:2:2, NMC 8:1:1, NCA or Co_3O_4. Lung sections were stained with Sirius red (magnification 200x).

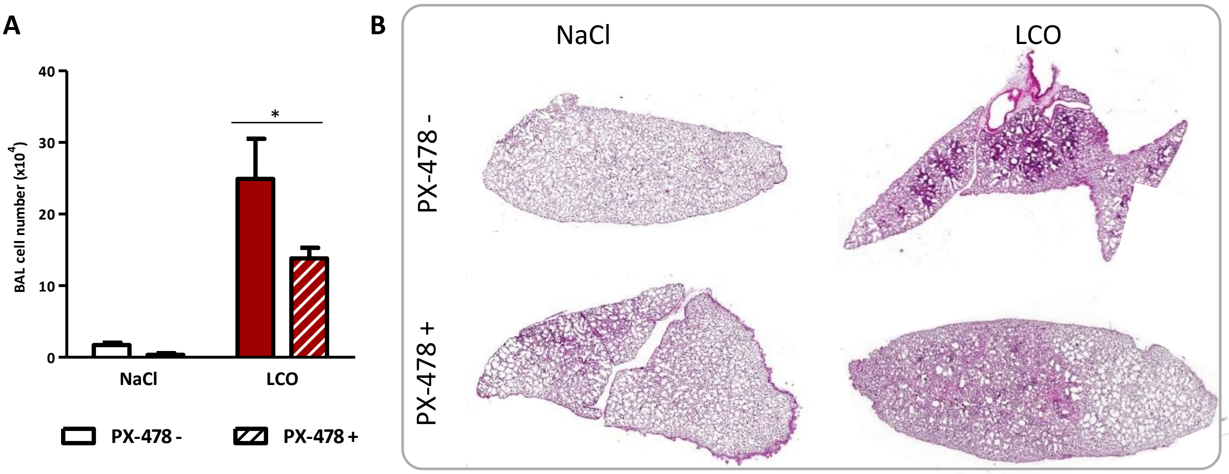

Figure S3: HIF-1α drives lung inflammation induced by LCO. C57BL/6Jrj mice were treated with an oro-pharyngeal aspiration of NaCl (control) or 2 mg LCO. Mice were treated with i.p. injections of 20 mg/kg bw/d PX-478 or with the vehicle (saline solution with 10 % DMSO) at day -1, 1 and 2. Mice were euthanized after 3 days. Inflammatory cell infiltration was assessed in the BAL (a). Lung sections were stained with HE (magnification 10x) (b). *P < 0.05, **P < 0.01 and ***P < 0.001 (t-test between PX-478 - and + mice for each condition, N = 1, n = 5, means ± SEM).

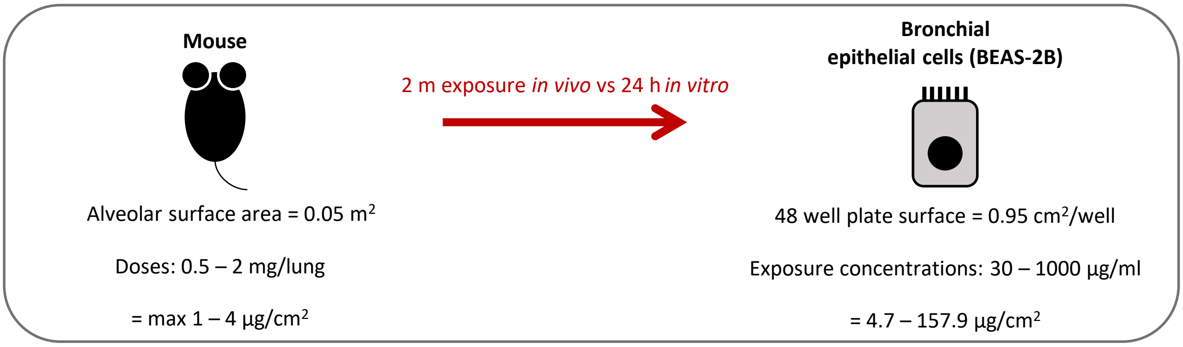

Figure S4: Comparison of *in vivo* and *in vitro* doses.
